# Parallel Local–Global Feature Extraction and Cross-Attention Gated Fusion for Network Intrusion Detection

**DOI:** 10.3390/s26196016

**Published:** 2026-09-23

**Authors:** Tinghui Huang, Yu Wang, Yuming Qin

**Affiliations:** 1 School of Computer Science and Information Security, Guilin University of Electronic Technology, Guilin 541004, China; 2 School of Electronic Information, Guilin University of Electronic Technology, Beihai 536000, China; wy782271124@163.com (Y.W.); 18784154142@163.com (Y.Q.)

**Keywords:** network intrusion detection, local–global feature extraction, feature fusion, cross-attention, gating mechanism

## Abstract

With the rapid advancement of network technology and the Internet of Things (IoT), massive, high-dimensional traffic data pose significant challenges to Network Intrusion Detection Systems (NIDS). Existing deep learning methods face two major limitations: (1) Insufficient feature extraction: single models struggle to concurrently capture local and global features, while serial hybrid architectures are prone to feature information loss. (2) Superficial feature fusion: existing strategies overlook deep interactions between features and lack adaptive filtering mechanisms to eliminate redundant information. To address these issues, a Parallel Local–Global Feature Extraction (PLGFE) module is constructed, incorporating a dual-stream architecture with parallel Temporal Convolutional Network (TCN) and Bidirectional Long Short-Term Memory (BiLSTM) branches. This module utilizes TCN’s dilated convolutions and BiLSTM’s bidirectional memory units to concurrently extract complementary local and global features. The Cross-Attention Gating (CAG) module integrates cross-attention and gating mechanisms for feature fusion: the former enables deep interaction between heterogeneous features, while the latter adaptively selects key features and suppresses redundant interaction features through gated weights. Experiments on the CICIDS2017 and CICIoV2024 datasets demonstrate the proposed method achieves multi-classification accuracies of 99.85% and 99.64%, with F1-Scores of 97.03% and 97.64%, respectively. Comparisons and ablation experiments confirm its competitive detection performance and component effectiveness.

## 1. Introduction

Driven by the rapid evolution of internet technology and the proliferation of emerging paradigms such as the Internet of Things (IoT) and cloud computing, modern network environments are becoming increasingly complex and heterogeneous [1]. While this high degree of interconnectedness significantly accelerates societal digital transformation, it also fosters complex and evolving cybersecurity threats, with various malicious attack methods exhibiting trends toward automation, stealth, and diversification [2]. Therefore, research on NIDS, as a critical defense for maintaining cybersecurity, has become particularly crucial. Traditional intrusion detection methods primarily rely on signature-based detection mechanisms and extensive domain expertise [3]. However, these traditional methods face severe challenges when confronted with the new characteristics of modern network traffic, including massive volume, high dimensionality, and dynamic changes [4].

In response to the rigidity of signature-based detection mechanisms and the bottleneck of manual domain expertise, researchers have increasingly applied machine learning (ML) algorithms to intrusion detection. These aim to identify network attacks by automatically learning behavioral patterns from historical traffic data. Many classical machine learning algorithms have demonstrated effectiveness in specific scenarios by focusing on classifier ensembling and feature space optimization. For instance, ensemble and hybrid methods, such as combining Support Vector Machines (SVM) with k-Nearest Neighbors (KNN) [5] or utilizing Genetic Algorithms (GA) to optimize feature selection prior to SVM classification [6], have been proposed to significantly enhance classification adaptability. Furthermore, tree-based and probabilistic models—including Random Forest equipped with feature subset elimination [7] and Hidden Naive Bayes (HNB) integrating discretization techniques [8]—have shown superior performance in mitigating noisy data interference and improving detection rates for specific attacks. However, traditional machine learning methods inherently belong to shallow learning and face a critical bottleneck: their performance heavily relies on manual feature engineering and domain-specific prior knowledge. When confronted with massive and complex raw traffic data, manually extracted features often prove subjective and incomplete, struggling to capture deep nonlinear correlations within the data. This limits the model’s generalization capability and detection accuracy in large-scale network environments.

To overcome these inherent limitations of traditional machine learning methods, deep learning has made significant strides in intrusion detection in recent years, leveraging its powerful automatic feature extraction and nonlinear pattern recognition capabilities. Deep learning models can automatically learn high-level abstract features directly from raw traffic data through multi-layer nonlinear mappings, effectively solving the modeling challenges of large-scale, high-dimensional data. Convolutional Neural Networks (CNNs) have demonstrated exceptional performance in cybersecurity, particularly in network intrusion detection. For instance, Qazi et al. [9] proposed a deep learning system based on one-dimensional convolutional neural networks (1D-CNN) tailored to the temporal characteristics of network traffic data. This method employs Random Undersampling (RUS) techniques to effectively mitigate the severe class imbalance in datasets, achieving efficient detection of multiple attack types (e.g., DDoS, PortScan) without complex feature engineering. Addressing the low detection rates for minority-class attacks and high false alarm rates in existing methods, Ren et al. [10] proposed a hierarchical CNN-Attention model named CANET. This model combines CNN with attention mechanisms to form a CABlock for extracting local spatio-temporal features. It innovatively introduces the EqualizationLossv2 function to balance the weights of minority class samples, significantly reducing false positives while improving overall detection accuracy. Parallel to CNNs, Recurrent Neural Networks (RNNs) and their variants possess efficient sequence data processing capabilities, achieving notable progress in network intrusion detection. For instance, addressing security protection needs in IoT fog computing environments, Almiani et al. [11] designed a cascaded intrusion detection model based on Deep Recurrent Neural Networks (DeepRNN). This model utilizes the Kalman Filter algorithm for weight updates to accelerate convergence and employs a multi-layer filtering structure to effectively enhance detection robustness against attacks such as DoS and Probe. Furthermore, to address challenges posed by high-dimensional data and imbalanced distributions, Devendiran et al. [12] proposed a hybrid deep learning system named Dugat-LSTM. This system first employs the Extended Synthetic Sampling (ExSyn) method to balance the dataset, utilizes Kernel-Assisted Principal Component Analysis (KerPCA) for feature extraction, and selects the optimal feature subset via the Chaotic Honey Badger Optimization (CHbO) algorithm. Finally, it combines a gated attention dual LSTM network to achieve high-precision classification of complex network attacks. In summary, these studies demonstrate that deep learning exhibits significant advantages over shallow models in enhancing detection accuracy and robustness.

Although the aforementioned deep learning methods have made remarkable progress in network intrusion detection, single models struggle to capture both local and global features simultaneously, while serial hybrid architectures are highly prone to information loss. Moreover, heterogeneous feature fusion remains superficial, as existing fusion strategies overlook deep interactions between local and global features and lack adaptive filtering mechanisms to eliminate redundant information. Therefore, this paper proposes a network intrusion detection method to address these issues. The main contributions of this work are summarized as follows:

- We design a Parallel Local–Global Feature Extraction (PLGFE) module, adapting the parallel dual-stream architecture specifically to the characteristics of network traffic. This framework utilizes parallel TCN and BiLSTM branches to capture local and global features, respectively. It effectively overcomes the limited feature capture scope of single models while preventing the loss of information inherent in traditional serial architectures, thus providing a foundational framework for the subsequent fusion module.
- We propose a Cross-Attention Gating (CAG) module to effectively generate highly discriminative fusion features. This module integrates cross-attention mechanisms with adaptive gating units: the cross-attention facilitates deep interactions between local and global features, while the gating units adaptively filter key information, thereby suppressing redundant noise.
- We conduct comprehensive experimental validation on the CICIDS2017 and CICIoV2024 datasets. The results demonstrate that the proposed method achieves superior detection performance compared to existing state-of-the-art methods. Furthermore, detailed ablation studies validate the individual contribution and effectiveness of each proposed component.

The remainder of this work is organized as follows: Section 2 reviews related works; Section 3 introduces the overall architecture of the proposed method and details its key components; Section 4 describes the datasets and experimental design while reporting ablation and comparative results; and Section 5 concludes the paper.

## 2. Related Work

### 2.1. Local and Global Feature Extraction

The core task of deep learning models in intrusion detection is the automatic extraction of discriminative features. However, network traffic data simultaneously contains both local features and global features, and a single type of neural network often struggles to extract both types effectively.

Regarding local feature extraction, CNNs and their variants excel at capturing correlations between adjacent packets, known as local features, owing to their local receptive field mechanism [13]. This characteristic renders them highly sensitive to transient high-frequency traffic bursts, such as those observed in DDoS attacks. However, constrained by the limited size of convolutional kernels, these models often struggle to capture long-range dependencies across the entire sequence. Conversely, RNNs and their variants maintain state information via memory units, designed to capture long-term evolutionary trends, known as global features [14]. This capability is crucial for identifying prolonged attacks like slow scanning or persistent penetration. Yet when processing high-dimensional traffic, they often overlook underlying local details.

To extract different feature types, some hybrid models adopt serial structures. For instance, Xie et al. [15] proposed a hierarchical spatio-temporal feature model (HSTF) for HTTP-based Trojan detection. Their approach employs a CNN to process raw packets represented as two-dimensional matrices, extracting spatial features via convolution and pooling operations. These features are then combined with packet-level statistics and subsequently fed into a Long Short-Term Memory (LSTM) network to capture long-term dependencies. Similarly, Wu et al. [16] proposed the Active Deep Q-Network (ADQN) framework for zero-day attack detection. This method utilizes a 1D-CNN to process preprocessed traffic, extracting spatial features through local connections and weight sharing. These features are then reshaped and passed to a Bidirectional LSTM (BiLSTM) to analyze temporal correlations. Nevertheless, this serial design creates an inherent information bottleneck. The initial extraction phase often causes the irreversible loss of raw information, providing degraded representations to the subsequent stage and ultimately constraining the overall detection accuracy.

To overcome information loss from serial structures, more researchers have shifted focus to parallel structures, aiming to independently and comprehensively preserve local and global information. Parallel structures process raw inputs through two independent channels, enabling separate extraction of local and global features. For instance, Li and Xu [17] proposed the L2-BITCN-CNN model for multi-classification based on spatio-temporal feature fusion. They adopted a parallel dual-stream network architecture, utilizing a bidirectional temporal convolutional network (BiTCN) and a CNN for independent feature extraction. The BiTCN branch processes one-dimensional byte sequences via a dual-layer attention mechanism to capture bidirectional temporal traffic features. Concurrently, the CNN branch processes transformed two-dimensional numerical matrices using multi-dimensional convolutional kernels to extract spatial packet features. Finally, the model fuses the extracted spatio-temporal feature vectors via concatenation and feeds them into a Softmax classifier for recognition. Lin et al. [18] applied a parallel framework (MSSTFF-S) to non-intrusive load monitoring. Their spatial branch integrates an enhanced ConvNeXt module with Efficient Channel Attention (ECA) to capture subtle state changes, while a BiGRU branch concurrently learns temporal dependencies. A multi-scale module further refines these features before fusion, ensuring accurate state identification. Song et al. [19] proposed the TGA model, which leverages a parallel combination of TCN and BiGRU. Specifically, the TCN branch focuses on extracting local temporal features, whereas the BiGRU branch captures global dependencies within the sequence, ensuring the independent capture of diverse behavior patterns. Similarly, targeting complex IoT environments, Wang et al. [20] developed an intelligent digital twin attack recognition method. Their model runs CNN and BiLSTM networks in parallel: the CNN extracts spatial attack features from traffic-derived images (RGB/TAM), while the BiLSTM extracts bidirectional temporal dependencies to construct comprehensive feature representations.

However, while parallel architectures effectively extract local and global features, they shift the primary challenge toward the need for effective feature fusion strategies. Because these dual-stream models process data in parallel, they isolate local and global features into separate feature spaces. Acquiring these features alone is insufficient. Without an effective fusion mechanism to construct comprehensive fusion features, these architectures cannot fully exploit their complementary advantages, ultimately restricting the overall detection performance.

### 2.2. Feature Fusion Strategy

In current parallel IDS frameworks, the predominant fusion method remains simplistic. For instance, the studies in [17,18] adopted a concatenation approach. This method directly joins feature vectors, ignoring the differences in numerical distribution and semantic representation between local and global features. To enhance feature fusion, some studies have begun exploring the application of attention mechanisms. For instance, Song et al. [19] introduced self-attention following concatenation, while Wang et al. [20] proposed an attention-based weighted summation directly to the parallel branches. Although these methods offer partial optimization, achieving deep feature interaction remains difficult. Critically, self-attention is primarily designed to capture internal dependencies within a single sequence, while weighted summation essentially remains a linear superposition. Neither approach facilitates deep interactions between the separate feature spaces, resulting in a lack of communication and complementarity between the local and global features.

To address these limitations, cross-attention mechanisms offer a more effective approach. Unlike self-attention, cross-attention enables one branch’s features to “query” another branch’s features—a mechanism that has demonstrated exceptional heterogeneous feature interaction capabilities in multimodal learning. For instance, the Vision-and-Language BERT (ViLBERT) model proposed by Lu et al. [21] introduces dual-stream co-attentional transformer layers, enabling deep information exchange between visual and linguistic streams while maintaining independent processing. Shi et al. [22] designed the Decoupled Cross modal Phrase-Attention (DCPA) network, which employs cross-attention to establish precise, fine-grained semantic correspondences between visual phrases and textual phrases, effectively addressing heterogeneity in cross-modal retrieval. Meanwhile, Zhu et al. [23] proposed the Cross-modal Hierarchical Attention for Recognizing Malicious Memes (CHARM) framework, which employs a cross-modal guidance module to facilitate dynamic information exchange between modalities via bidirectional attention flows, enabling the acute detection of implicit semantic conflicts. Inspired by these approaches, integrating cross-attention into IDS compels the model to establish connections between local and global features, thereby achieving deep feature interaction. Furthermore, considering that features after deep interaction may contain redundant information, introducing a gating mechanism enables effective filtering. This adaptively preserves discriminative information for classification while suppressing irrelevant interaction noise. In summary, combining cross-attention and adaptive gating mechanisms is essential for constructing highly discriminative fusion features in parallel models.

Consequently, to overcome the limitations of simple concatenation and self-attention, it is a critical requirement to develop a feature fusion strategy that integrates cross-attention for deep interactions between local and global features with gating mechanisms for adaptive suppression of interaction-induced redundancy.

## 3. Methods

### 3.1. Overall Framework

To effectively address the limitations of single models and serial architectures in extracting local and global features, and to overcome the insufficient feature interaction and redundancy interference during feature fusion, this work proposes a novel intrusion detection network. As illustrated in Figure 1, the overall workflow of the proposed network comprises three core modules: the Parallel Local–Global Feature Extraction (PLGFE) module, the Cross-Attention Gating (CAG) module, and the classifier module. Specifically, the preprocessed traffic data is first fed into the PLGFE module, which extracts local and global features through a parallel dual-stream architecture. Subsequently, these features are passed into the CAG module for feature fusion. This module establishes deep feature interaction and adaptively removes redundant features generated during the interaction phase, generating highly discriminative fusion features. Finally, these features are fed into the classifier module, comprising standard fully connected layers and a Softmax activation function, to map the fused representations to the label space and output the final classification results.

### 3.2. Parallel Local–Global Feature Extraction (PLGFE) Module

To comprehensively capture local and global features in network traffic data, the proposed network employs a dual-stream parallel feature extraction module, as illustrated in Figure 1. Since the input consists of flow-based tabular records, the 1D feature vector of each record is mapped into a sequence based on the arrangement of its statistical attributes. Through this sequential mapping, the module is designed to learn the latent local and global correlations and dependencies between features, rather than actual temporal traffic patterns. Unlike serial architectures that may suffer from information loss, the parallel architecture processes the preprocessed data independently. Specifically, the module comprises two branches working in parallel: a TCN branch utilized to extract local features, and a BiLSTM branch employed to capture global features. The PLGFE module ensures that both local and global features are preserved without mutual interference, providing a complete feature set for the subsequent feature fusion.

#### 3.2.1. TCN Branch for Local Feature Extraction

TCN [24] serves as the core component for extracting local features within the PLGFE module. Compared to the 1D-CNN, the TCN incorporates a dilated causal convolution mechanism. This design enables an exponential expansion of the receptive field without increasing network depth or parameter complexity.

As illustrated in Figure 2, dilated causal convolution expands the coverage area by inserting zeros between kernel weights. To quantify the efficiency of the TCN in capturing local features, its receptive field is analyzed. Assuming the network comprises *L* layers of dilated causal convolutions with a kernel size of *k*, and the dilation factor for the *i* layer is set to $d_{i}=2^{i}$, the maximum effective historical receptive field, denoted as *R*, is calculated as shown in Equation (1):

(1) $$R=1+\sum_{i=0}^{L-1}\left(k-1\right)\cdot d_{i}=1+\left(k-1\right)\cdot \left(2^{L}-1\right)$$

As derived from the equation, the receptive field *R* of the TCN grows exponentially with the number of layers *L*, whereas that of a standard 1D-CNN (where $d_{i}=1$) increases only linearly. This implies that TCN requires significantly fewer layers to capture a wider range of local information, thereby efficiently extracting local features.

Figure 3 illustrates the specific structure of the TCN branch. The proposed TCN branch implements a 4-layer architecture. Within this branch, the output channels of the four successive layers are sequentially set to 64, 64, 128, and 128, respectively, with the kernel size set to k=3. Additionally, the dropout rate of 0.1 is applied to prevent overfitting. Furthermore, to mitigate the vanishing gradient problem and preserve low-level feature information, the TCN branch incorporates standard residual connections. Specifically, the input is processed through a 1×1 convolution to align channel dimensions before being added element-wise to the deep features extracted via dilated causal convolutions. After processing through these four layers, the branch generates a local feature matrix, denoted as $F_{\mathrm{local}}\in R^{128\times T}$ (where the sequence dimension *T* corresponds to the input feature dimension), thereby providing local features for the subsequent feature fusion.

#### 3.2.2. BiLSTM Branch for Global Feature Extraction

Considering that network traffic intrinsically encompasses not only local features but also global features, a BiLSTM [25] branch is deployed in parallel with the TCN branch, as illustrated in Figure 4. Although BiGRU offers computational efficiency, it combines the functions of the cell state and hidden state into a single hidden state representation. Consequently, this absence of an independent cell state limits its capability to model long-range dependencies when processing high-dimensional vectors. To address this limitation, the proposed BiLSTM branch maintains an independent cell state to effectively decouple long-term information retention from the step-wise hidden state output, thereby significantly enhancing the retention of long-range dependencies across the entire feature space. In terms of the specific configuration, this branch implements a 2-layer stacked architecture with hidden sizes of 64 and 128, respectively. Through a bidirectional recurrent architecture, the model achieves a comprehensive modeling of both forward and backward dependencies. To ensure numerical stability and prevent gradient anomalies during deep feature propagation, a Layer Normalization (LayerNorm) operation is applied after each BiLSTM layer. Ultimately, this branch generates a global feature matrix, denoted as the $F_{\mathrm{global}}\in R^{T\times 256}$. This matrix is then input into the CAG module for feature fusion with $F_{\mathrm{local}}$.

### 3.3. Cross-Attention Gating (CAG) Module

To effectively integrate the local and global features ($F_{\mathrm{global}}$ and $F_{\mathrm{local}}$) extracted by the PLGFE module, this work proposes the CAG module. Existing feature fusion methods, such as simple concatenation and self-attention, often fail to capture the mutual dependencies between local and global features and are susceptible to irrelevant interaction noise. To overcome these limitations, the CAG module establishes a feature fusion strategy that not only facilitates deep feature interaction but also adaptively filters out redundant features generated during the cross-attention phase. As illustrated in Figure 5, the CAG module consists of three key components: the Feature Projection Layer, the Cross-Attention Block, and the Adaptive Gating Unit.

#### 3.3.1. Feature Projection Layer

The local features $F_{\mathrm{local}}$ and global features $F_{\mathrm{global}}$ extracted by the preceding PLGFE module, after applying global average pooling, possess distinct feature dimensions, denoted as $D_{\mathrm{local}}$ and $D_{\mathrm{global}}$, respectively (where $D_{\mathrm{local}}\neq D_{\mathrm{global}}$). However, the subsequent Cross-Attention Block and Adaptive Gating Unit involve element-wise operations and matrix multiplications that strictly require the input features to be aligned within a unified latent space.

To address this dimensional mismatch and semantic gap, the Feature Projection Layer is introduced to map these heterogeneous features into a unified latent space with dimension $D_{\mathrm{fusion}}$ (set to 128), as illustrated in the left panel of Figure 5. Specifically, a composite projection function consisting of a linear layer, LayerNorm, and a ReLU activation function is employed. LN is applied to stabilize the feature distributions and prevent gradient instability, while the ReLU activation introduces non-linearity and promotes feature sparsity by suppressing negative noisy activations. The projection process is formulated as Equation (2):

(2) $$\begin{array}{cc} F_{\mathrm{local}}^{\mathrm{proj}} & =\mathrm{ReLU}\left(\mathrm{LN}\left(F_{\mathrm{local}}\cdot W_{L}+b_{L}\right)\right)\\ F_{\mathrm{global}}^{\mathrm{proj}} & =\mathrm{ReLU}\left(\mathrm{LN}\left(F_{\mathrm{global}}\cdot W_{G}+b_{G}\right)\right) \end{array}$$

where $W_{L}\in R^{D_{\mathrm{local}}\times D_{\mathrm{fusion}}}$ and $W_{G}\in R^{D_{\mathrm{global}}\times D_{\mathrm{fusion}}}$ denote the learnable weight matrices, and $b_{L},b_{G}\in R^{D_{\mathrm{fusion}}}$ represent the corresponding bias terms. Through this projection, both feature representations are aligned into the same dimensional space, thereby establishing a strictly compatible mathematical foundation for the subsequent cross-attention and gating mechanisms.

#### 3.3.2. Cross-Attention Block

While the local and global features have been mapped to a unified latent space, they remain as independent representations. To implement the deep interaction between local and global features, the Cross-Attention Block is designed, as illustrated in the central region of Figure 5.

Unlike standard self-attention mechanisms that compute correlations within a single sequence, this module employs a multi-head cross-attention mechanism with h=4 parallel heads. The mechanism enables one feature branch to “query” the opposite branch to retrieve complementary contextual information. Specifically, the Query (*Q*) matrices are derived from the primary branch, while the Key (*K*) and Value (*V*) matrices are projected from the reciprocal branch. This structural design forces the model to focus on the complementary information that is most highly correlated with the current branch’s representations.

To be specific, two symmetric interaction processes are formulated: Local-Querying-Global and Global-Querying-Local.

Local-Querying-Global (Enriching Local Features): To incorporate global contextual information into the local feature representation, the projected local features $F_{\mathrm{local}}^{\mathrm{proj}}$ are linearly transformed to generate the Queries ($Q_{\mathrm{local}}$), while the projected global features $F_{\mathrm{global}}^{\mathrm{proj}}$ are utilized to yield the Keys ($K_{\mathrm{global}}$) and Values ($V_{\mathrm{global}}$). The linear projection operations are formulated as Equation (3):

(3) $$Q_{\mathrm{local}}=F_{\mathrm{local}}^{\mathrm{proj}}\cdot W_{Q,L},\;K_{\mathrm{global}}=F_{\mathrm{global}}^{\mathrm{proj}}\cdot W_{K,G},\;V_{\mathrm{global}}=F_{\mathrm{global}}^{\mathrm{proj}}\cdot W_{V,G}$$

where $W_{Q,L},\;W_{K,G},\;W_{V,G}\in R^{D_{\mathrm{fusion}}\times D_{\mathrm{fusion}}}$ denote the learnable weight matrices. The interaction features $F_{\mathrm{local}}^{\mathrm{cross}}$ are then computed using the scaled dot-product attention, formulated as Equation (4):

(4) $$F_{\mathrm{local}}^{\mathrm{cross}}=\mathrm{Softmax}\left(\frac{Q_{\mathrm{local}}{\left(K_{\mathrm{global}}\right)}^{T}}{\sqrt{D_{\mathrm{fusion}}}}\right)V_{\mathrm{global}}\cdot W_{O,L}$$

where $W_{O,L}\in R^{D_{\mathrm{fusion}}\times D_{\mathrm{fusion}}}$ represents the output projection matrix. The Softmax function is applied along the key dimension to normalize the attention weights. Through this process, $F_{\mathrm{local}}^{\mathrm{cross}}$ effectively aggregates global contextual information that exhibits a high correlation with the specific local patterns.

Global-Querying-Local (Refining Global Features): Symmetrically, to incorporate the local patterns into the global feature representation, the roles of the inputs are reversed. The projected global features $F_{\mathrm{global}}^{\mathrm{proj}}$ are linearly transformed to generate the Queries ($Q_{\mathrm{global}}$), while the projected local features $F_{\mathrm{local}}^{\mathrm{proj}}$ are utilized to yield the Keys ($K_{\mathrm{local}}$) and Values ($V_{\mathrm{local}}$). Following a symmetrical computational process to Equations (3) and (4), this reciprocal interaction yields the interaction features $F_{\mathrm{global}}^{\mathrm{cross}}$. Through this process, $F_{\mathrm{global}}^{\mathrm{cross}}$ effectively aggregates specific local patterns that exhibit a high correlation with the global contextual information.

#### 3.3.3. Adaptive Gating Unit

Although the Cross-Attention Block successfully facilitates deep feature interaction between the local and global features, not all the interaction features are beneficial. Directly integrating these interaction features into the original projected features without selective filtering may introduce redundancy or irrelevant noise, potentially confusing the model and degrading detection performance. To address this limitation, the Adaptive Gating Unit is designed, as illustrated in the right panel of Figure 5.

It calculates an adaptive gating coefficient based on the relative importance of the interaction features with respect to the original projected features. Taking the local branch as an instance, the original projected features $F_{\mathrm{local}}^{\mathrm{proj}}$ and the interaction features $F_{\mathrm{local}}^{\mathrm{cross}}$ are first concatenated along the feature dimension to form a comprehensive representation. This concatenated representation is then fed into a gating network, consisting of a linear layer, LayerNorm, and a Sigmoid activation function, to generate the adaptive gating coefficient $\alpha_{\mathrm{local}}$, formulated as Equation (5):

(5) $$\alpha_{\mathrm{local}}=\sigma \left(\mathrm{LN}\left(W_{g,L}\cdot \left[F_{\mathrm{local}}^{\mathrm{proj}};F_{\mathrm{local}}^{\mathrm{cross}}\right]+b_{g,L}\right)\right)$$

where σ denotes the Sigmoid activation function, [·;·] represents the concatenation operation, $W_{g,L}\in R^{2D_{\mathrm{fusion}}\times D_{\mathrm{fusion}}}$ denotes the learnable weight matrix, and $b_{g,L}\in R^{D_{\mathrm{fusion}}}$ is the corresponding bias term. Crucially, LayerNorm is applied prior to the activation function. This design stabilizes the input feature distribution, preventing the Sigmoid function from prematurely entering saturation zones during early training stages, thereby ensuring the gating mechanism can accurately evaluate their relative importance.

With the learned coefficient $\alpha_{\mathrm{local}}$, a residual gating fusion is performed. The original projected features serve as the backbone, while the interaction features are treated as a controllable refinement term, yielding the refined local features ${\tilde{F}}_{\mathrm{local}}$, formulated as Equation (6):

(6) $${\tilde{F}}_{\mathrm{local}}=F_{\mathrm{local}}^{\mathrm{proj}}+\alpha_{\mathrm{local}}⊙F_{\mathrm{local}}^{\mathrm{cross}}$$

where ⊙ denotes element-wise multiplication. In this formulation, if $\alpha_{\mathrm{local}}\approx 0$, the module suppresses the redundant interaction features, preserving the original projected features; if $\alpha_{\mathrm{local}}\approx 1$, the module actively integrates the interaction features. Symmetrically, the refined global features, denoted as ${\tilde{F}}_{\mathrm{global}}$, are computed as Equation (7):

(7) $${\tilde{F}}_{\mathrm{global}}=F_{\mathrm{global}}^{\mathrm{proj}}+\alpha_{\mathrm{global}}⊙F_{\mathrm{global}}^{\mathrm{cross}}$$

Finally, the refined features from both branches are concatenated to form the final fused representation $F_{\mathrm{fusion}}$, which is subsequently fed into the classifier module, formulated as Equation (8):

(8) $$F_{\mathrm{fusion}}=\left[{\tilde{F}}_{\mathrm{local}};{\tilde{F}}_{\mathrm{global}}\right]$$

## 4. Experimental Results and Analysis

### 4.1. Datasets

To comprehensively evaluate the detection performance and adaptability of the proposed method across distinct traffic scenarios, two representative public datasets are selected for experimentation: CICIDS2017 [26], widely used in traditional network intrusion detection, and CICIoV2024 [27], tailored for modern Internet of Vehicles (IoV) environments.

CICIDS2017 Dataset: CICIDS2017 is a benchmark dataset released by the Canadian Institute for Cybersecurity (CIC), designed to reflect the true characteristics and diversity of modern network traffic. This dataset contains five days of benign traffic alongside various common attack patterns. The CSV format version of this dataset, comprising approximately 2.8 million network flow records, was utilized in the experiments. Each record consists of 78 traffic features and one label. The dataset covers multiple attack categories including Web Attacks, Brute Force, Denial of Service (DoS), Botnet, PortScan, and Infiltration, among others. Compared to older datasets like KDD-99 or NSL-KDD, the attack patterns in CICIDS2017 better align with the current internet threat landscape.

CICIoV2024 Dataset: With the proliferation of IoV technology, attacks targeting vehicle networks are increasing. To validate model effectiveness in emerging network environments, the CICIoV2024 dataset is adopted. This dataset simulates diverse communication scenarios within IoV environments, capturing traffic characteristics during interactions between internal vehicle networks (e.g., CAN bus) and external networks. The dataset comprises 12 features: 9 traffic features and 3 label features. It includes 5 specific attack types, primarily categorized into DoS and Spoofing attacks. Spoofing attacks encompass Gas Spoofing, RPM Spoofing, Speed Spoofing, and Steering Wheel Spoofing.

In real-world traffic, benign samples constitute the vast majority, while attack samples (especially certain specific spoofing or penetration attacks) are relatively scarce. Both datasets face the challenge of data imbalance. Detailed sample distributions across categories are shown in Table 1 and Table 2.

### 4.2. Experimental Setup and Evaluation Metrics

Experiments were conducted on a machine equipped with a 13th Gen Intel^®^ Core^TM^ i7-13650HX 2.60 GHz CPU, 16 GB RAM, and an NVIDIA GeForce RTX 4060 Laptop GPU with 8 GB memory. Model development was performed in Python using PyTorch 2.1.1; metric calculations were executed with Scikit-learn, and data processing utilized Pandas and NumPy.

During model training, key hyperparameters were iteratively tuned to achieve rapid convergence and optimal performance. The core parameter settings were established as follows: the maximum number of training epochs was set to 100, and the batch size was set to 2048, with an early stopping mechanism triggered if validation loss failed to improve over 20 consecutive epochs. The Adam optimizer was selected with an initial learning rate of 0.01, employing the ReduceLROnPlateau learning rate scheduler with a minimum learning rate of $1\times 10^{-6}$. CrossEntropyLoss was adopted as the loss function to measure the discrepancy between the predicted probabilities and the true labels.

To comprehensively and objectively quantify the detection performance, Accuracy, Precision, Recall, and F1-Score are employed as evaluation metrics, calculated using the macro-average strategy [28]. This mechanism assigns equal weight to each category by calculating metrics independently for each class and taking their unweighted mean. This strategy fairly reflects the model’s performance on minority attack classes, ensuring that their evaluation is not overshadowed by majority classes with massive sample sizes [29]. Consequently, it provides a more authentic and rigorous assessment of the proposed method’s overall effectiveness on heavily imbalanced datasets.

### 4.3. Data Preprocessing

Due to noise, varying feature scales, and severe class imbalance in raw network traffic data, directly feeding it into deep learning models can lead to training instability. To enhance data quality and ensure the reliability of model evaluation, the preprocessing pipeline was executed:

Step 1: Data Cleaning and Consolidation. To eliminate noise, corrupted records containing missing values (NaN), infinite values (Infinity), and duplicates were first removed, followed by label encoding for non-numeric features. For CICIDS2017, a domain-driven class consolidation strategy was applied (Table 3), largely aligning with the dataset’s original conceptual attack families. Specifically, FTP-Patator and SSH-Patator are merged into “Brute Force” due to shared dictionary-based cracking behaviors. Distinct web attack subtypes form “Web Attack”, and four DoS variants (Hulk, GoldenEye, slowloris, Slowhttptest) are consolidated into “DoS” given their shared application-layer resource exhaustion patterns. In contrast, “DDoS” remains separate since its distributed nature generates distinct volumetric traffic compared to single-source DoS. “PortScan” is maintained independently as a reconnaissance activity, fundamentally distinct from exploitation behaviors. Lastly, Heartbleed and Infiltration are preserved individually, as their unique exploitation mechanisms do not align with other major families. The CICIoV2024 dataset retained its original structure.

Step 2: Dataset Partitioning. Following the initial cleaning, the dataset was divided into training, validation, and test sets using a stratified random sampling strategy with a 60%:20%:20% ratio. This ensures that the original class proportions are maintained across all subsets.

Step 3: Feature Standardization. Given the vast variation in the value ranges of network traffic features, Min-Max normalization was applied. The scaler was fitted exclusively on the training set to establish the [0,1] mapping intervals, which were then used to transform the validation and test sets.

Step 4: Class Balancing. To address severe class imbalance, a hybrid resampling strategy combining the Synthetic Minority Oversampling Technique (SMOTE) and Random Undersampling (RUS) was implemented exclusively on the training set. The sample counts before and after class balancing are shown in Figure 6. The validation and test sets retained their original imbalanced distributions without any resampling intervention, preserving that the models are evaluated in real-world network environments.

### 4.4. Classification Performance on the CICIDS2017 and CICIoV2024 Datasets

To comprehensively visualize the multi-class classification capability of the complete model under complex traffic conditions, we generated count and normalized confusion matrices for the CICIDS2017 and CICIoV2024 test sets, as illustrated in Figure 7 and Figure 8, respectively. As observed from the diagonal distributions in the normalized confusion matrices in Figure 7b and Figure 8b, the model achieves high prediction accuracy across both multi-classification tasks. Furthermore, the count confusion matrices depicted in Figure 7a and Figure 8a confirm that the model maintains low false negative rates across most categories, although containing false positives in certain minority attack classes remains challenging.

To provide a more granular evaluation of the model’s prediction capabilities and investigate specific misclassifications, we present the detailed per-class performance metrics alongside the overall evaluation criteria in Table 4 and Table 5. As presented, the proposed model achieves an impressive overall accuracy of 99.85% and 99.64% on the CICIDS2017 and CICIoV2024 datasets, respectively, complemented by macro-average F1-scores of 97.03% and 97.64%.

While the model demonstrates excellent overall performance, the detailed analysis reveals that the precision for a few specific attack categories is relatively lower. For instance, in the CICIDS2017 dataset, the Bot class exhibits a precision of 0.6477. Similarly, in the CICIoV2024 dataset, the Speed class yields a precision of 0.8306. These localized discrepancies indicate that although the model successfully extracts local and global composite features to maintain near-perfect recall rates, distinguishing these highly confusable attack samples from benign traffic still inherently produces a certain degree of false positives. Recognizing these specific misclassifications highlights the ongoing challenges in network intrusion detection and points to potential areas for future feature optimization.

### 4.5. Ablation Experiments

To validate the effectiveness and necessity of each key component in the proposed model, two sets of progressive ablation experiments are designed: the first aims to evaluate the advantages of the PLGFE module at the feature extraction stage; the second focuses on verifying the performance improvement of the CAG module compared to existing fusion strategies. Specifically, four distinct configurations were constructed: Model-1 (TCN-only) employs a standalone TCN to focus solely on local features; Model-2 (BiLSTM-only) retains only the BiLSTM to extract global features; Model-3 (Serial TCN-BiLSTM) adopts a serial structure where TCN outputs are fed sequentially into the BiLSTM; and Model-4 implements the proposed parallel PLGFE module, employing simple concatenation for feature fusion to strictly isolate the impact of the extraction architectures.

The experimental results are shown in Table 6. It can be observed that the F1-Scores of Model-1 and Model-2, both employing a single network architecture, are 91.70% and 90.76%, respectively, which are noticeably lower than those of the serial and parallel architectures. This empirically validates the premise that a singular network architecture struggles to comprehensively capture the complex traffic patterns of modern network attacks. Furthermore, the serial structure (Model-3) underperforms the parallel structure (Model-4) in terms of overall performance. Notably, while Model-3 achieves the highest Recall (99.67%), this comes at the cost of diminished Precision. This provides direct experimental evidence that in the serial architecture, sequentially feeding deep convolutional features into the BiLSTM forces the model to be overly sensitive to anomalies, yet crucial discriminative information is distorted or lost during the deep mapping, leading to more false positives. In contrast, the parallel architecture of the proposed PLGFE module enables the two branches to perform feature mapping in independent, non-interfering subspaces. This design simultaneously accommodates the extraction of both local and global features, effectively balancing Precision and Recall, thereby elevating the F1-Score to 93.28%.

Following the validation of the PLGFE module’s superiority, the second set of ablation experiments further investigates the practical effectiveness of the cross-attention and adaptive gating mechanisms within the CAG module. Accordingly, three variants of feature fusion strategies were constructed: Model-5 introduces a standard self-attention mechanism after the PLGFE module, focusing solely on the internal dependencies within the concatenated high-dimensional feature vector; Model-6 incorporates a cross-attention mechanism to guide deep feature interaction between the TCN and BiLSTM branches, but removes the adaptive gating unit; and finally, the proposed complete model, which simultaneously incorporates both the cross-attention and gating mechanisms.

As indicated by the ablation results in Table 7, compared to Model-4 utilizing the simple concatenation operation, the models incorporating attention mechanisms consistently yield performance gains. Notably, Model-6 (cross-attention without the adaptive gating unit) outperforms Model-5 (self-attention) by a margin of 0.90% in the F1-Score. This result reflects the mechanistic differences between the two attention mechanisms when processing homologous multi-scale features: self-attention primarily mines internal dependencies within a single representation space, which tends to cause feature homogenization; whereas cross-attention facilitates deep interaction between local and global features through a directed querying mechanism, thereby enhancing the overall fusion capability while preserving their distinct representational strengths. Ultimately, the complete model equipped with the adaptive gating unit achieves the highest F1-Score of 97.03%. It is worth observing that models without the gating unit (such as Model-5 and Model-6) exhibit slightly higher Recall. This is because ungated attention mechanisms aggressively retain all correlated features, making the model highly sensitive to attacks but concurrently susceptible to false positives. The proposed CAG module effectively addresses this by intentionally trading a marginal fraction of Recall to filter out redundant interaction noise. This adaptive suppression drastically boosts Precision to 95.28%, demonstrating that the gating mechanism is crucial for achieving the optimal comprehensive classification performance. Crucially, these progressive performance improvements—from 92.14% in a conventional serial setup (Model-3) and 93.28% via simple concatenation (Model-4), to 97.03% in the fully integrated architecture—demonstrate that the specific combination of the PLGFE and CAG modules creates a strong synergistic effect rather than a simple modular addition. The parallel architecture effectively prevents the information loss inherent in serial designs to provide a complete feature foundation, while the subsequent cross-attention and gating units facilitate deep feature interactions and suppress redundant noise.

### 4.6. Comparative Experiments

To objectively evaluate the detection capability of the proposed method in the current field of network intrusion detection, this section conducts a detailed comparison with mainstream state-of-the-art (SOTA) methods. The selected baseline models encompass classical machine learning frameworks, ensemble learning algorithms, and mainstream deep learning tabular models from recent years, aiming to evaluate the proposed method across multiple dimensions. To ensure fairness and maintain uniform testing conditions, all selected baseline models were reproduced and evaluated within the same experimental framework. Table 8 presents the comprehensive performance comparison between the proposed method and various representative models across multiple evaluation metrics.

On the CICIDS2017 dataset, the proposed method demonstrates strong competitiveness in overall performance, confirming its effectiveness in processing traditional complex network traffic. Specifically, while baseline models such as LightGBM and FT-Transformer achieve high classification accuracy (99.90% and 99.86%, respectively) and recall (99.43% and 99.71%, respectively), their precision scores remain relatively limited (89.06% and 88.98%, respectively). Even compared to the parallel TGA model, which achieves a higher precision of 93.08%, the proposed method still significantly improves precision to 95.28% while maintaining highly competitive accuracy (99.85%) and recall (99.50%). In terms of the F1-Score, which balances precision and recall, the proposed method achieved the optimal performance of 97.03%, outperforming the TGA model (95.41%), LightGBM (93.34%), FT-Transformer (92.86%), and XGBoost (89.58%). Additionally, the proposed method maintains an extremely low false positive rate (0.02%) and a high PR-AUC (98.02%), validating its superior comprehensive detection capability.

On the emerging IoV dataset CICIoV2024, the adaptability of the model to recent IoV traffic patterns was tested. The accuracy and recall of the proposed method reached 99.64% and 98.44%, respectively. Compared to the strongest baseline models, the TGA model and TabNet, which obtained 99.62% and 99.61% accuracy, respectively, alongside a shared 98.44% recall, the proposed method demonstrates a competitive edge in accuracy while maintaining the same optimal recall. In terms of precision, the proposed method achieved 97.18%, marginally outperforming the TGA model (97.05%) and TabNet (97.01%), and significantly exceeding FT-Transformer (95.64%) and XGBoost (94.93%). Consequently, for the F1-Score, the proposed method reached the best performance of 97.64%, surpassing the TGA model (97.57%), TabNet (97.54%), FT-Transformer (96.77%), and XGBoost (96.49%). Furthermore, the proposed method maintains an extremely low false positive rate (0.06%) and a high PR-AUC (99.31%). Notably, the proposed method maintains highly stable overall performance even in complex IoV environments. This demonstrates that the PLGFE and CAG modules can effectively adapt to the intricate communication environments within the IoV, showcasing significant application potential in this domain.

### 4.7. Computational Cost and Real-Time Performance Analysis

Computational efficiency is a practical factor for real-world NIDS deployment. To ensure a fair comparison, the proposed method and six baseline models were evaluated under identical hardware and software environments. We measured the number of trainable parameters, GPU memory footprint, inference latency, real-time throughput, as well as training time and convergence speed. The results are summarized in Table 9.

As shown in Table 9, traditional tree-based models like XGBoost achieve high throughputs (>514 K samples/s) and low inference latency. However, as discussed in previous sections, they yield relatively lower detection performance on complex attacks. On the other hand, deep tabular models like FT-Transformer and TabNet exhibit significantly lower throughputs (<38 K samples/s). Specifically, although TabNet possesses the smallest parameter size (0.03 M) and lowest GPU memory footprint (26.59 MB), its inference latency (0.0264 ms) remains higher than that of the proposed method. Meanwhile, the TGA model demonstrates strong real-time inference speed (86,762 samples/s), but requires a considerably larger GPU memory footprint (679.88 MB).

In contrast, the proposed method provides a practical balance between computational cost and detection capability. It achieves a real-time throughput of over 67,700 samples/s, nearly doubling the processing speed of TabNet despite a moderate increase in model size (0.63 M) and memory footprint (476.64 MB). Furthermore, considering the practical necessity of offline model retraining in dynamic network environments, the proposed architecture demonstrates excellent computational efficiency during the training phase. It requires only 34.0 s per epoch, making it the fastest among all deep learning arrays, notably outperforming the strongest baseline, TGA (45.0 s/epoch). Although it reaches optimal convergence at Epoch 43, the significantly reduced per-epoch overhead ensures a highly competitive total retraining duration. These results indicate its high efficiency and feasibility for practical network deployment.

### 4.8. Statistical Reliability Analysis

To rigorously validate the statistical reliability of the proposed method and eliminate the potential performance bias introduced by random initialization, we conducted 5 independent trials. Specifically, the proposed method and the baseline models were retrained and evaluated across 5 different random seeds. Table 10 reports the mean (μ) and standard deviation (σ) of the comprehensive evaluation metrics, including Accuracy, Precision, Recall, and F1-Score.

As demonstrated in Table 10, the proposed method consistently exhibits extremely low standard deviations across all four core evaluation metrics during the 5 independent runs. This confirms that the overall detection performance is highly stable, rather than an artifact of a specific random seed. To further substantiate the statistical reliability, we conducted a paired *t*-test. We selected the TGA model (94.82 ± 0.66%) for this comparison, as it represents the strongest related baseline in our benchmarks. The paired *t*-test comparing the F1-Scores yielded a *T*-statistic of 4.5919 and a *p*-value of 0.0101 (p<0.05). This statistical analysis confirms that the performance improvement achieved by the proposed architecture is statistically significant, demonstrating its effectiveness in mitigating class imbalance.

### 4.9. Discussion and Limitations

While the proposed PLGFE and CAG modules demonstrate competitive overall performance, it is essential to objectively acknowledge several statistical and architectural limitations that may impact their practical usability in real-world NIDS deployments.

Statistical Fragility of Extreme Minority Classes: First, the headline Macro-F1 metrics can be easily inflated by extreme minority classes. In CICIDS2017, attacks like Heartbleed and SQL Injection have only single-digit instances in the test set. Because macro-averaging assigns equal weight to all classes, perfect predictions on these tiny subsets count exactly as much toward the overall 97.03% Macro-F1 as the Benign class. Furthermore, this fragility is exacerbated by the SMOTE-RUS resampling mechanism. For extremely rare attacks, its interpolation may generate tightly clustered synthetic samples, leading the model to overfit these localized points rather than generalizing to the true distribution. Thus, perfect scores (e.g., 1.0000 for Heartbleed) should be interpreted cautiously. Second, while the macro-average strategy provides a balanced theoretical evaluation by treating all attack categories equally, it overlooks the varying operational costs of misclassification in practical NIDS deployment. In real-world environments, the cost of missing a critical attack (false negative) significantly outweighs the cost of a false alarm (false positive). To address this limitation, future implementations should incorporate cost-sensitive evaluation matrices and apply targeted threshold tuning to prioritize high-risk threats.

Precision Failures and Alert Fatigue: Specific precision failures in certain attack categories highlight challenges for security applications. As shown in Table 4, the Bot category in CICIDS2017 exhibits a relatively low precision (0.6477) despite its high recall (0.9665). In a deployed NIDS, such a high proportion of false positives can translate into alert fatigue for security analysts. Similarly, Table 5 illustrates confusion between RPM and Speed spoofing attacks in the CICIoV2024 dataset. The model misroutes 9.27% of RPM samples to Speed (yielding an RPM recall of 0.9073), which inevitably drags the Speed precision down to 0.8306 despite its perfect recall.

Challenges with Highly Similar Attacks: These specific misclassifications reveal a practical limitation of the Cross-Attention and Gating (CAG) module. While the CAG mechanism works well for most attack types, it struggles to separate attacks that share very similar traffic patterns, such as RPM and Speed. Because the data features of these two attacks heavily overlap, the module is prone to misclassifying them. To address this in future work, we need to perform a more detailed feature analysis to find the exact differences between these similar attacks. Additionally, adjusting the model training to strictly penalize false positives would help reduce such false alarms.

Model Interpretability: Finally, while the proposed architecture achieves competitive detection performance, explicitly visualizing the high-dimensional decision-making process of the cross-attention and gating mechanisms remains a challenge. Therefore, exploring dedicated Explainable AI (XAI) techniques to provide intuitive visualizations of this feature fusion process represents a crucial direction for our future work.

## 5. Conclusions

To address the challenges of insufficient local and global feature extraction and superficial heterogeneous feature fusion in existing NIDS, a network intrusion detection method is proposed in this paper. First, the PLGFE module is designed, utilizing a dual-stream architecture with parallel TCN and BiLSTM branches to concurrently extract both local and global features. Second, to overcome the limitations of existing fusion strategies, the CAG module is proposed. This module integrates cross-attention to facilitate deep interaction between local and global features, while employing an adaptive gating mechanism to effectively filter redundant interaction noise. Extensive experiments on the CICIDS2017 and CICIoV2024 datasets demonstrate that the proposed method achieves outstanding performance, with accuracies of 99.85% and 99.64%, and F1-Scores of 97.03% and 97.64%, respectively. These results, verified by comprehensive SOTA comparisons and detailed ablation experiments, firmly confirm that the combination of the PLGFE and CAG modules significantly enhances detection performance in complex network environments.

## Figures and Tables

**Figure 1:**
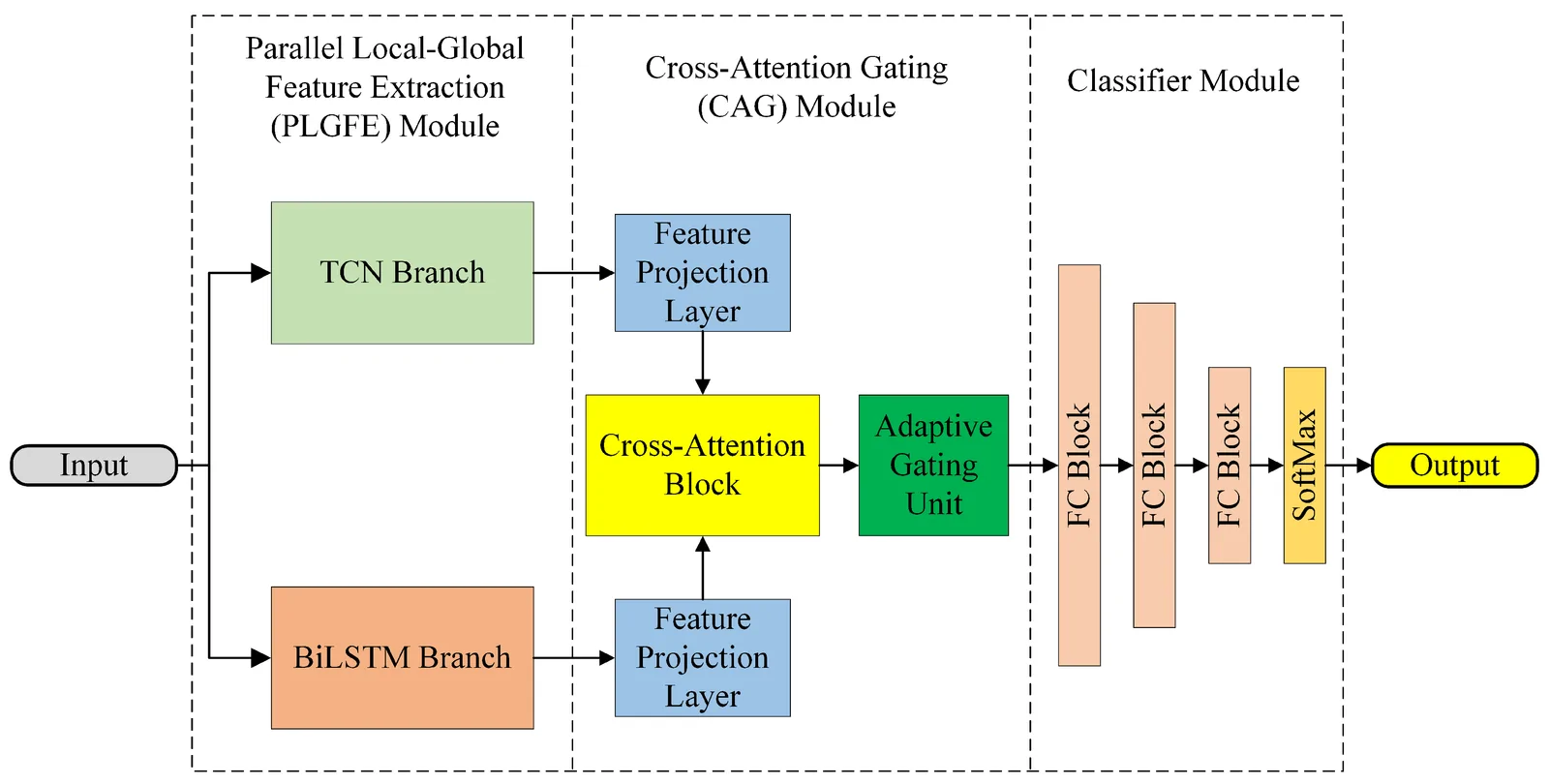
Overall architecture of the proposed method.

**Figure 2:**
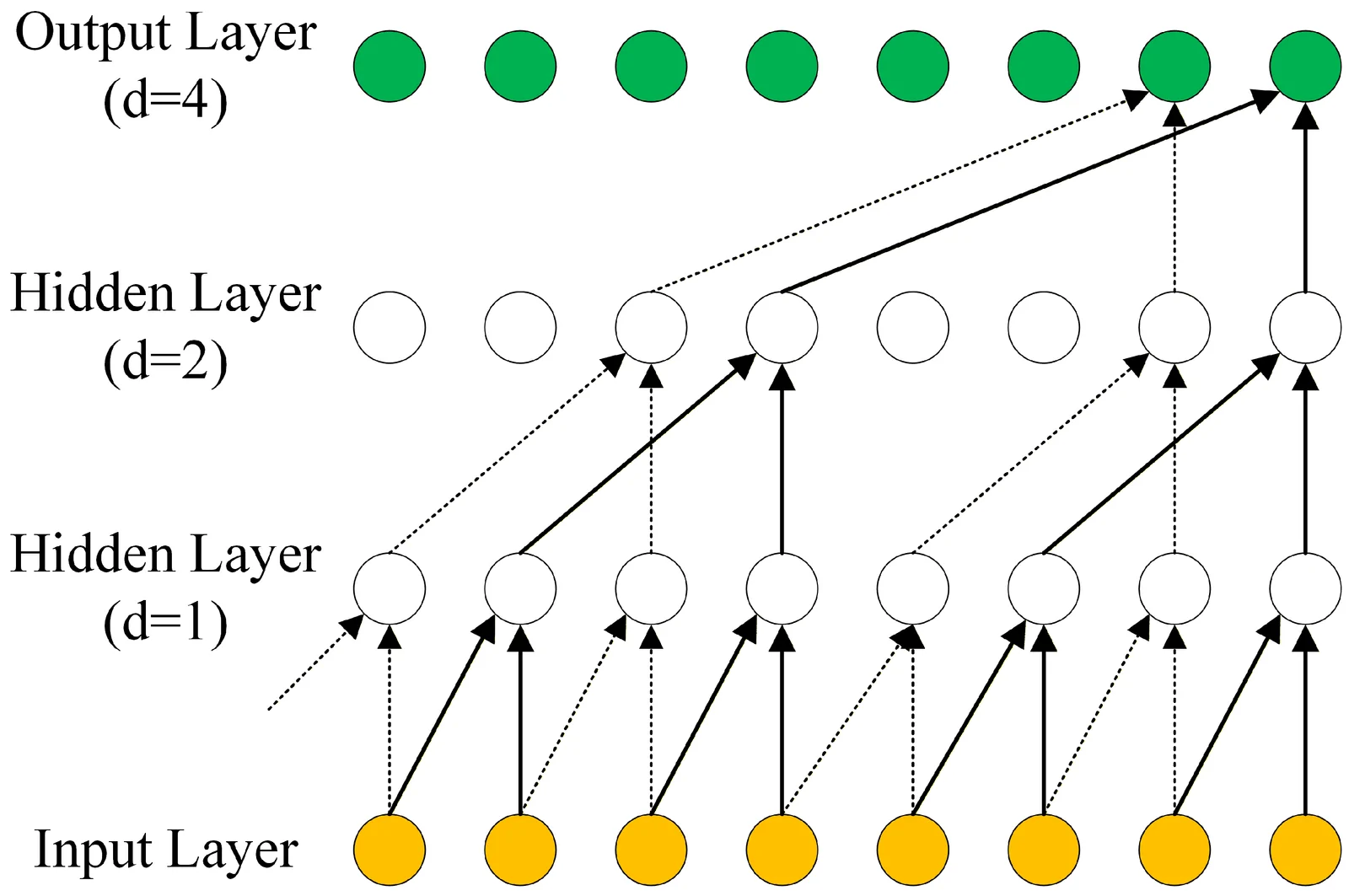
Structure of the dilated causal convolution module in TCN.

**Figure 3:**
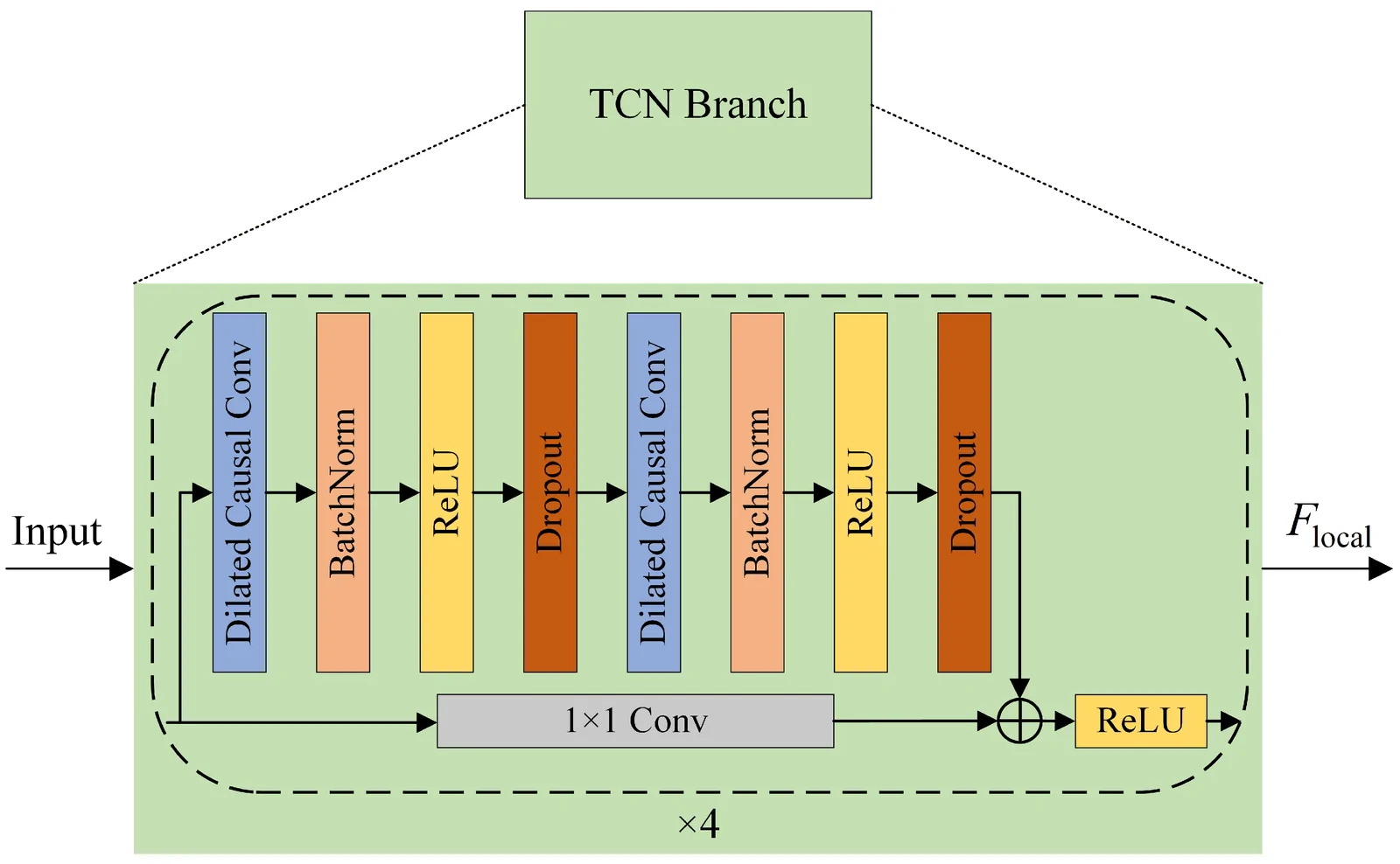
Structure of the TCN branch.

**Figure 4:**
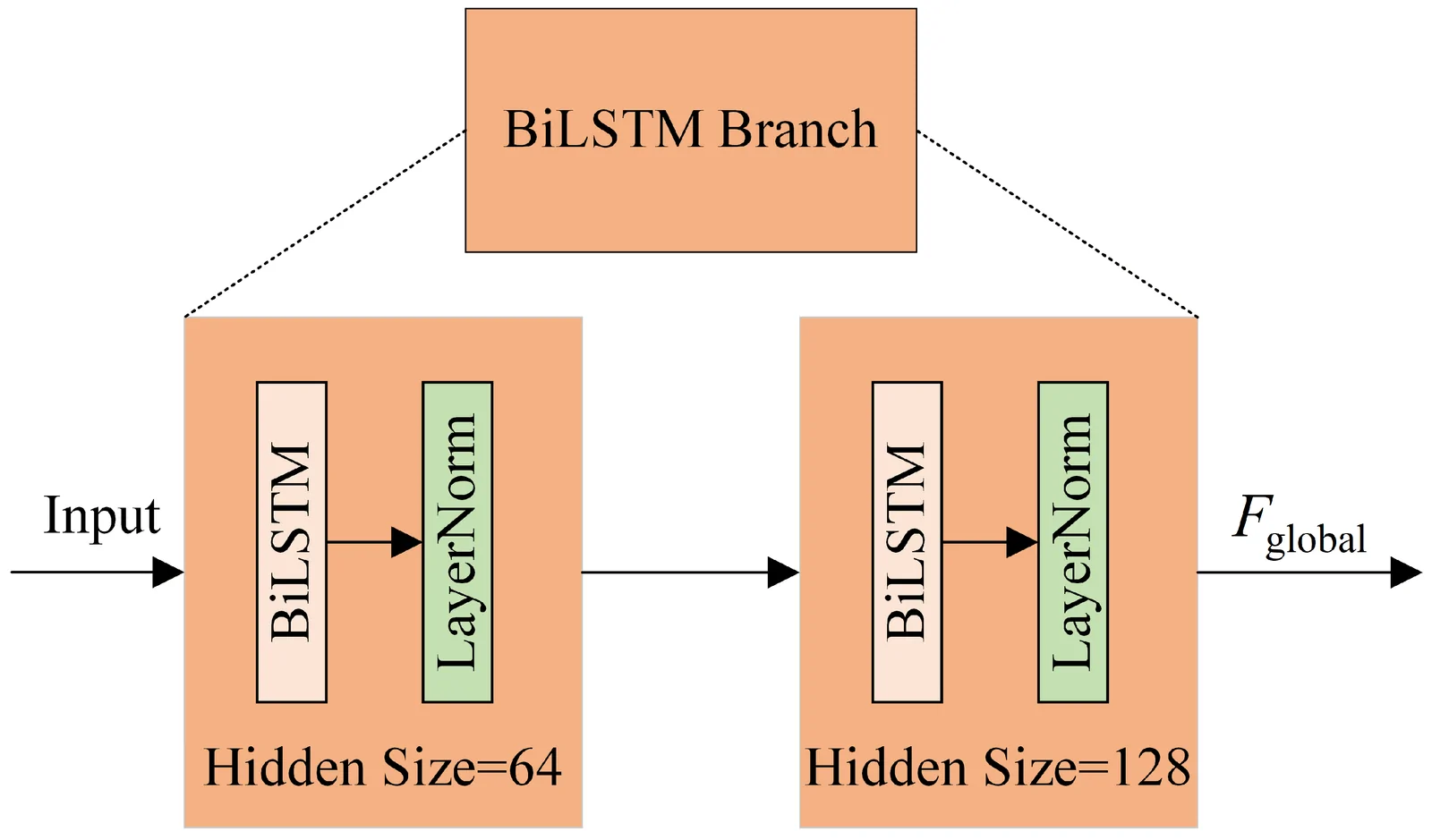
Structure of the BiLSTM branch.

**Figure 5:**
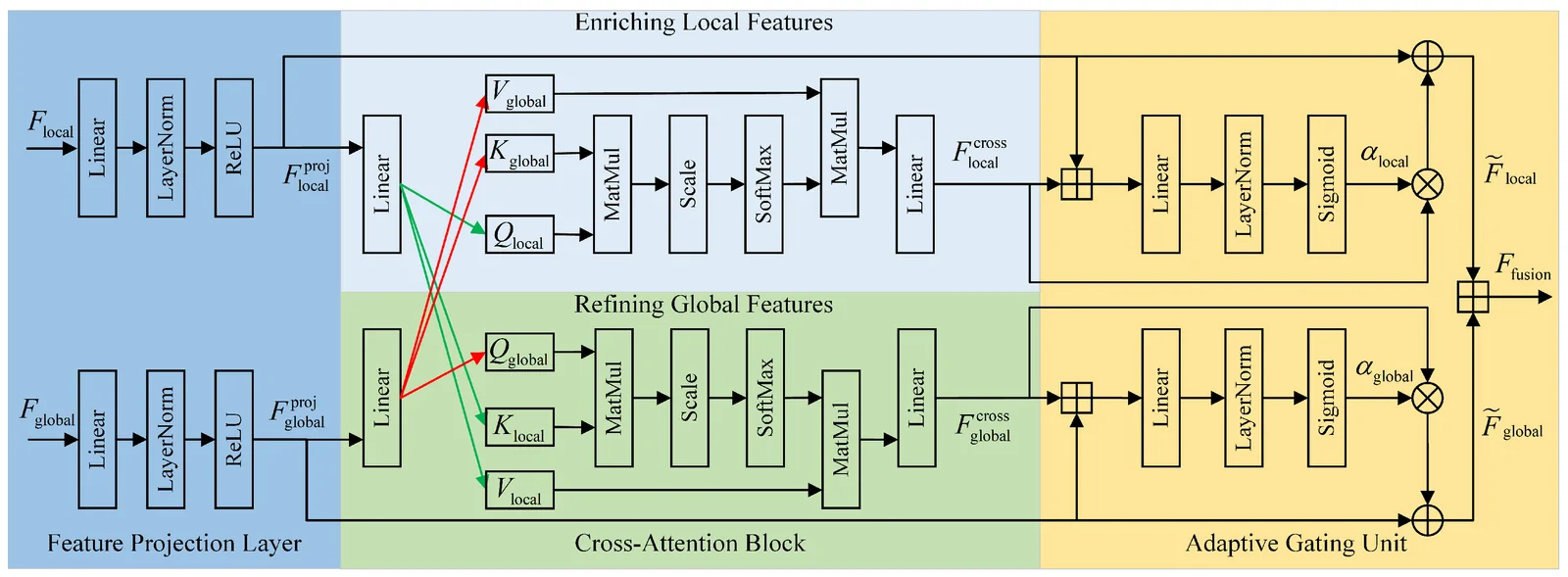
Structure of Cross-Attention Gating (CAG) module.

**Figure 6:**
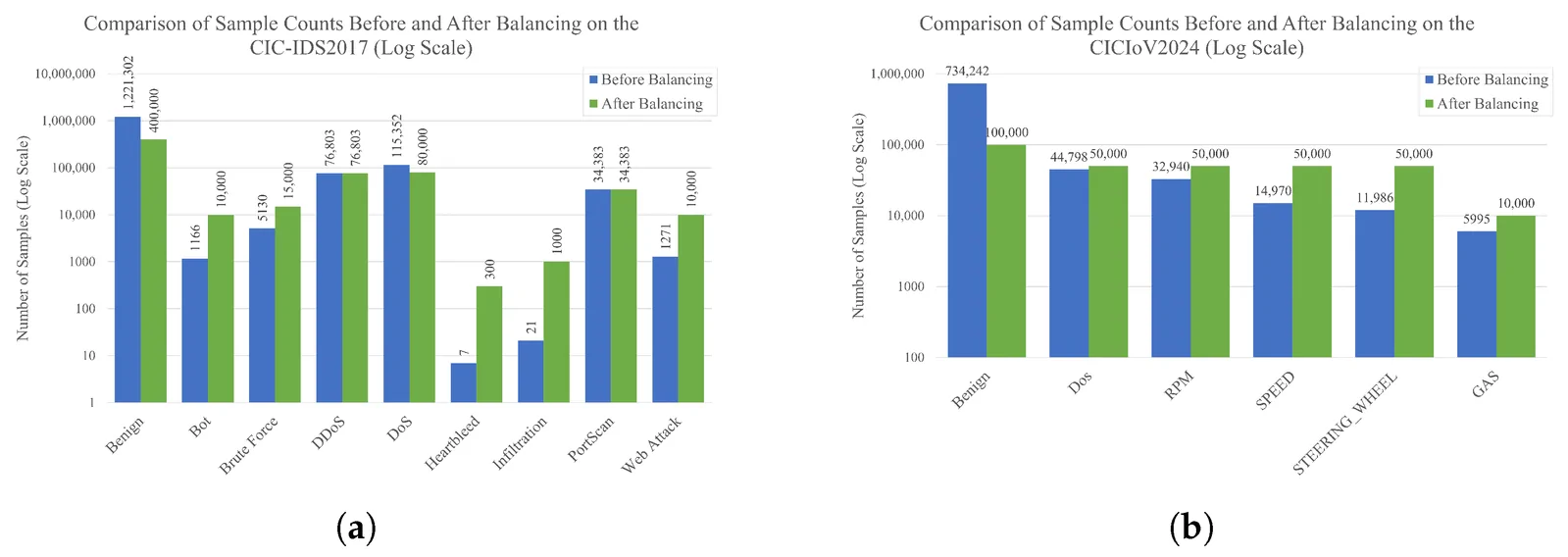
Comparison of sample counts before and after balancing (Log Scale). (**a**) CICIDS2017 dataset. (**b**) CICIoV2024 dataset.

**Figure 7:**
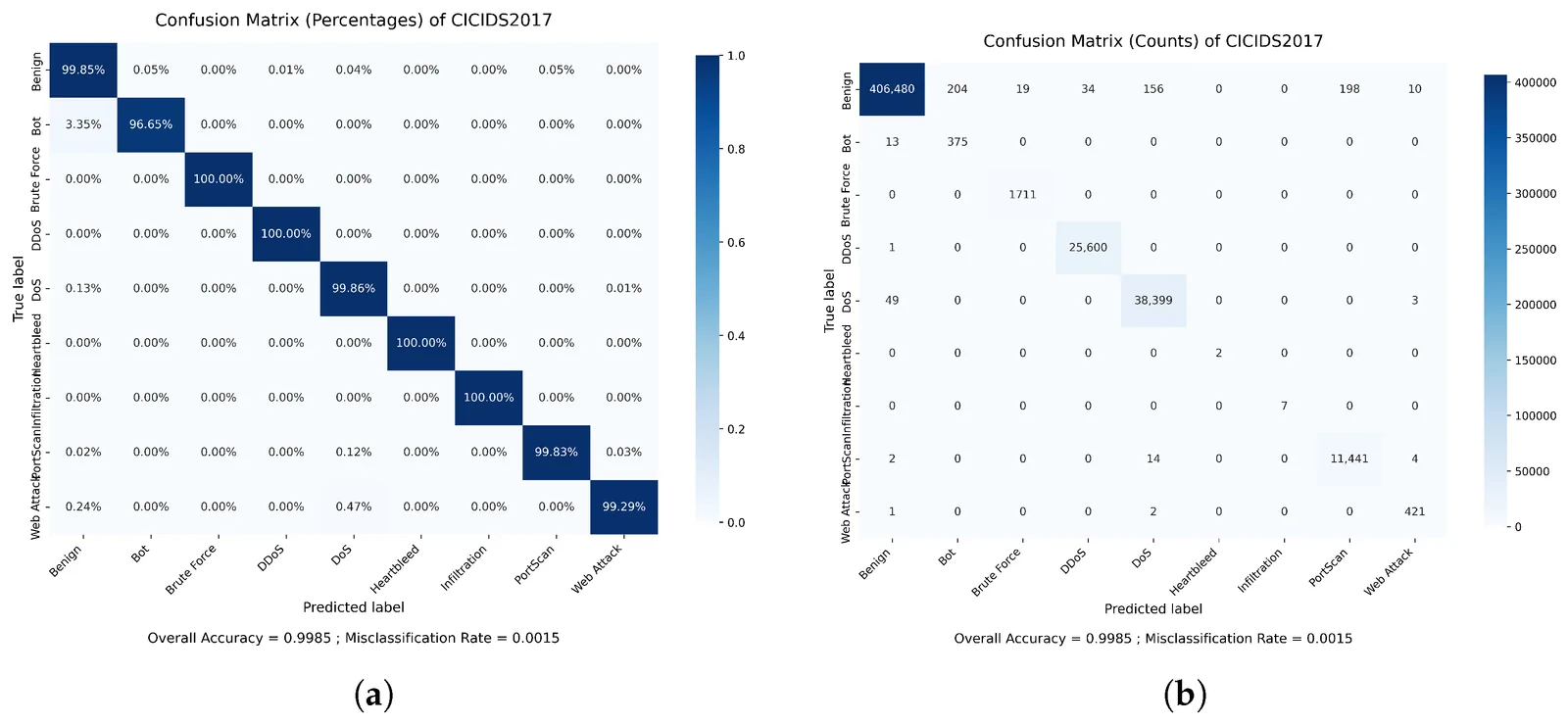
Confusion matrices on the CICIDS2017 dataset. (**a**) Count confusion matrix. (**b**) Normalized confusion matrix.

**Figure 8:**
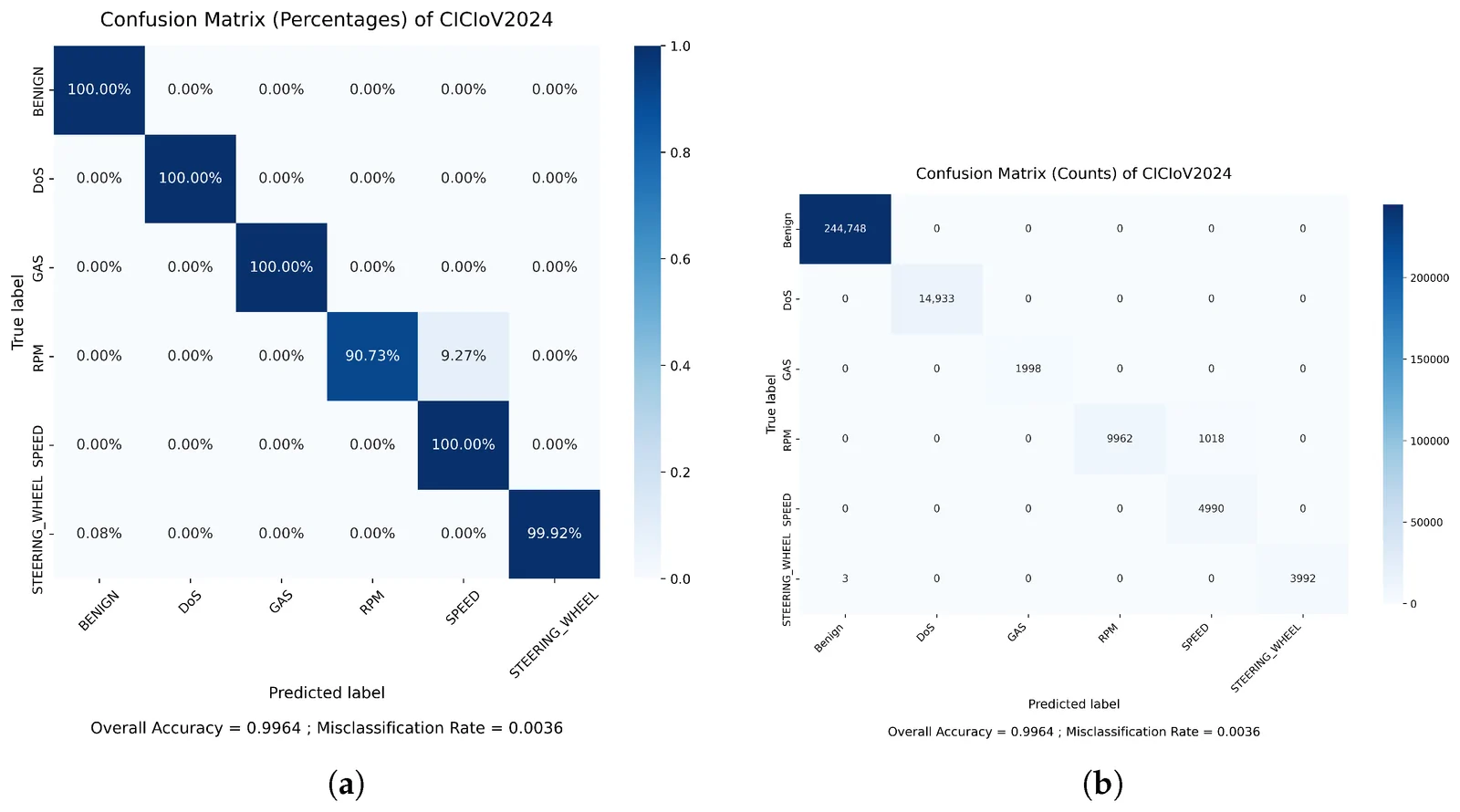
Confusion matrices on the CICIoV2024 dataset. (**a**) Count confusion matrix. (**b**) Normalized confusion matrix.

**Table 1:** Class distribution of the CICIDS2017 dataset.

Original Class	Sample Count	Percentage (%)
BENIGN	2,273,097	80.30
DoS Hulk	231,073	8.16
PortScan	158,930	5.61
DDoS	128,027	4.52
DoS GoldenEye	10,293	0.36
FTP-Patator	7938	0.28
SSH-Patator	5897	0.21
DoS slowloris	5796	0.20
DoS Slowhttptest	5499	0.19
Bot	1966	0.07
Web Attack Brute Force	1507	0.05
Web Attack XSS	652	0.02
Infiltration	36	<0.01
Web Attack Sql Injection	21	<0.01
Heartbleed	11	<0.01
Total	2,830,743	100.00

**Table 2:** Class distribution of the CICIoV2024 dataset.

Original Class	Sample Count	Percentage (%)
BENIGN	1,223,737	86.90
DoS	74,663	5.30
Spoofing-RPM	54,900	3.90
Spoofing-SPEED	24,951	1.77
Spoofing-STEERING_WHEEL	19,977	1.42
Spoofing-GAS	9991	0.71
Total	1,408,219	100.00

**Table 3:** Mapping details of class consolidation operations for the CICIDS2017 dataset.

Original Class	Operation	Final Class
BENIGN	Retain	BENIGN
DoS Hulk	Consolidate	DoS
PortScan	Retain	PortScan
DDoS	Consolidate	DDoS
DoS GoldenEye	Consolidate	DoS
FTP-Patator	Consolidate	Brute Force
SSH-Patator	Consolidate	Brute Force
DoS slowloris	Consolidate	DoS
DoS Slowhttptest	Consolidate	DoS
Bot	Retain	Bot
Web Attack Brute Force	Consolidate	Web Attack
Web Attack XSS	Consolidate	Web Attack
Infiltration	Retain	Infiltration
Web Attack Sql Injection	Consolidate	Web Attack
Heartbleed	Retain	Heartbleed

**Table 4:** Classification results on the CICIDS2017 dataset.

Class	Precision	Recall	F1-Score
Benign	0.9998	0.9985	0.9992
Bot	0.6477	0.9665	0.7756
Brute Force	0.9890	1.0000	0.9945
DDoS	0.9987	1.0000	0.9993
DoS	0.9955	0.9986	0.9971
Heartbleed	1.0000	1.0000	1.0000
Infiltration	1.0000	1.0000	1.0000
PortScan	0.9830	0.9983	0.9906
Web Attack	0.9612	0.9929	0.9768
Macro Average	0.9528	0.9950	0.9703
Overall Accuracy	0.9985		

**Table 5:** Classification results on the CICIoV2024 dataset.

Class	Precision	Recall	F1-Score
Benign	1.0000	1.0000	1.0000
DoS	1.0000	1.0000	1.0000
GAS	1.0000	1.0000	1.0000
RPM	1.0000	0.9073	0.9514
Speed	0.8306	1.0000	0.9074
Steering Wheel	1.0000	0.9992	0.9996
Macro Average	0.9718	0.9844	0.9764
Overall Accuracy	0.9964		

**Table 6:** Performance comparison of different feature extraction architectures.

Method	Architecture	Evaluation Metrics (%)			
		Accuracy	Precision	Recall	F1-Score
Model-1	TCN Only	99.72	89.57	96.08	91.70
Model-2	BiLSTM Only	99.78	85.93	99.25	90.76
Model-3	Serial TCN-BiLSTM	99.73	88.39	**99.67**	92.14
Model-4	PLGFE module (parallel)	**99.80**	**89.60**	99.63	**93.28**

The best results are highlighted in bold.

**Table 7:** Performance comparison of different feature fusion strategies.

Method	Fusion Strategy	Evaluation Metrics (%)			
		Accuracy	Precision	Recall	F1-Score
Model-4	Concatenation	99.80	89.60	99.63	93.28
Model-5	Self-Attention	99.83	92.46	**99.65**	95.26
Model-6	Cross-Attention w/o Gating	99.83	93.56	99.64	96.16
Proposed	CAG Module	**99.85**	**95.28**	99.50	**97.03**

The best results are highlighted in bold.

**Table 8:** Performance comparison on CICIDS2017 and CICIoV2024 datasets.

Dataset	Method	Accuracy	Precision	Recall	F1-Score	MCC	FPR	FNR	PR-AUC
CICIDS2017	Random Forest [30]	99.35	89.11	99.67	90.62	97.79	00.08	00.33	96.53
	LightGBM [31]	**99.90**	89.06	99.43	93.34	**99.64**	**00.02**	00.57	**99.18**
	XGBoost [32]	99.88	84.27	99.69	89.58	99.58	**00.02**	00.31	90.68
	TabNet [33]	99.60	77.55	99.11	82.70	98.60	00.08	00.89	94.54
	FT-Transformer [34]	99.86	88.98	**99.71**	92.86	99.53	**00.02**	**00.29**	99.00
	TGA Model [19]	99.82	93.08	99.53	95.41	99.38	00.03	00.47	98.17
	Proposed	99.85	**95.28**	99.50	**97.03**	99.49	**00.02**	00.50	98.02
CICIoV2024	Random Forest [30]	98.89	94.30	98.30	96.15	95.55	00.19	01.70	96.55
	LightGBM [31]	98.96	93.86	98.31	95.38	95.83	00.18	01.69	99.24
	XGBoost [32]	99.09	94.93	98.34	96.49	96.29	00.16	01.66	99.10
	TabNet [33]	99.61	97.01	**98.44**	97.54	98.39	00.07	**01.56**	99.27
	FT-Transformer [34]	99.20	95.64	98.36	96.77	96.75	00.14	01.64	99.24
	TGA Model [19]	99.62	97.05	**98.44**	97.57	98.43	00.07	**01.56**	99.30
	Proposed	**99.64**	**97.18**	**98.44**	**97.64**	**98.49**	**00.06**	**01.56**	**99.31**

The best results are highlighted in bold.

**Table 9:** Model complexity and real-time inference performance comparison.

Model	Parameters	GPU Memory	Latency	Throughput	Train Time	Convergence
	**(M)**	**(MB)**	**(ms/Sample)**	**(Samples/s)**	**(s/Epoch)**	**(Epochs)**
XGBoost [32]	N/A	CPU	0.0019	514,825.44	20.0 (Total)	N/A
LightGBM [31]	N/A	CPU	0.0163	61,465.05	80.3 (Total)	N/A
Random Forest [30]	N/A	CPU	0.0319	31,353.90	35.9 (Total)	N/A
TabNet [33]	0.03	26.59	0.0264	37,870.43	39.0	29
FT-Transformer [34]	0.91	513.33	0.0399	25,087.76	75.0	39
TGA [19]	0.55	679.88	0.0115	86,762.16	45.0	38
Proposed	0.63	476.64	0.0148	67,730.55	34.0	43

“N/A” indicates that the respective metrics (Parameters and Convergence epochs) are not applicable to non-parametric tree ensembles. “CPU” denotes that the traditional machine learning models were executed exclusively on the CPU. For these machine learning models, “Train Time” represents the total fitting time.

**Table 10:** Statistical reliability analysis (Mean ± Standard Deviation over 5 independent runs).

Model	Accuracy (%)	Precision (%)	Recall (%)	F1-Score (%)
XGBoost [32]	99.88 ± 0.00	84.27 ± 0.00	99.69 ± 0.00	89.58 ± 0.00
LightGBM [31]	99.89 ± 0.00	90.03 ± 1.98	99.26 ± 0.12	93.89 ± 1.18
Random Forest [30]	99.33 ± 0.02	85.94 ± 1.79	99.67 ± 0.02	88.69 ± 1.09
TabNet [33]	99.51 ± 0.21	74.29 ± 3.39	96.51 ± 2.46	79.03 ± 3.31
FT-Transformer [34]	99.85 ± 0.02	87.62 ± 1.54	99.71 ± 0.05	91.73 ± 1.34
TGA [19]	99.82 ± 0.01	92.16 ± 1.12	99.62 ± 0.90	94.82 ± 0.66
Proposed	99.88 ± 0.02	94.79 ± 0.90	99.63 ± 0.07	96.78 ± 0.53 *

* Indicates statistical significance (*p* < 0.05) against the strongest baseline (TGA) using a paired t-test.

## Data Availability

The original data presented in the study are openly available in the Canadian Institute for Cybersecurity (CIC) dataset repository. The CICIDS2017 dataset is available at https://www.unb.ca/cic/datasets/ids-2017.html (accessed on 19 September 2026), and the CICIoV2024 dataset is available at https://www.unb.ca/cic/datasets/iov-dataset-2024.html (accessed on 19 September 2026).
